# Acute cholestasis as uncommon onset of Kawasaki disease: a case report

**DOI:** 10.1186/s12876-020-01495-6

**Published:** 2020-10-28

**Authors:** Massimo Gallerani, Marco Pala, Fabio Fabbian, Alfredo De Giorgi

**Affiliations:** 1 Department of Medicine, Unit of Internal Medicine, Azienda Ospedaliera-Universitaria (AOU) of Ferrara, Via Aldo Moro 8, 44124 Cona, Ferrara, Italy; 2 Unit of Internal Medicine, Azienda Ospedaliera-Universitaria (AOU) of Ferrara, Ferrara, Italy; 3 Clinica Medica Unit, Department of Medical Sciences, Faculty of Medicine, Pharmacy and Prevention, University of Ferrara, Ferrara, Italy

**Keywords:** Kawasaki disease, Mucocutaneous lymph node syndrome, Acute cholestasis, Jaundice

## Abstract

**Background:**

Kawasaki disease (KD) or mucocutaneous lymph node syndrome is a vasculitis that mostly occurs in young children. Adult-onset KD (AKD) is rare and often misdiagnosed. Here we report a rare case of KD with cholestasis as principal symptom.

**Case presentation:**

A 43-year-old caucasian man was admitted to our hospital for high fever, lack of appetite related to nausea and vomiting, headache and significant malaise. Physical examination highlighted fever, increasing jaundice, bilateral laterocervical lymph nodes, erythema of the palms, and strikingly red lips and conjunctiva. The clinical course was complicated by arterial hypotension, tachycardia, decreasing haemoglobin, increasing acute phase reactants tests, and multiorgan failure. Due to cardiovascular instability the patient was admitted to the local Intensive Care Unit. Chest X-ray, abdominal ultrasound, chest and abdominal CT and Colangio Magnetic Resonance were normal. Jaundice was investigated and infections, autoimmune diseases or drugs adverse reactions, were excluded. Also coronary artery computed tomography was carried out excluding coronary artery aneurysms. Broad-spectrum antibiotics were not effective. After exclusion other possible conditions, diagnosis of KD was set. He was treated with high doses of corticosteroids and acetylsalicylic acid and clinical conditions as well as laboratory exams improved.

**Conclusions:**

This report dealing with an adult onset of atypical KD may be of benefit to physicians of various specialties, including primary care doctors, hospital internists, intensivists and gastroenterologists due to its peculiarities. It demonstrates that a case of prolonged fever unresponsive to antibiotics and related to cholestatic jaundice, oedema or erythema of the extremity associated with desquamation of feet and hands, and red eyes, may suggest atypical form of KD.

## Background

Kawasaki disease (KD) also defined mucocutaneous lymph node syndrome is an acute multi-systemic necrotizing vasculitis that affects middle and small sized vessels and occurs predominantly in young children. KD is more frequent (80% of cases) in young patients with an age between 6 months and 4 years. Being infrequent in Caucasian children, KD is very often misdiagnosed in European adult males [1–3]. As well as in other systemic diseases non-specific symptoms are common in the 10 days before diagnosis.

The suspect of KD is related to persistent fever (for more than 5 days), unresponsive to broad-spectrum antibiotics and with absence of proof of infection; clinical signs are: (1) oral lesions such as erythema of oral and pharyngeal mucosa, cracking or fissured of lips, strawberry tongue; (2) bilateral conjunctivitis without exudate; (3) polymorphous rash; (4) oedema or erythema of the extremity with a progressing to desquamation of feet and hands; (5) cervical lymphadenopathy usually unilateral (≥ 1.5 cm) [1–4].

These diagnostic criteria, valid for children, have not been validated in adults, in which incomplete forms are more frequent and related to possible severe complications due to under-diagnosis [1–4].

Several uncommon complications are related to inflammatory vascular lesions that affect not only coronary arteries but also all abdominal arteries, with aneurysm, calcification or stenosis [1–4]. Gastrointestinal and hepatic tracts involvement do not usually belong to KD; when it is present hepatic dysfunction, hepatomegaly, cholestatic hepatitis, hepatic artery aneurysm and hydrops of the gall bladder are the clinical features [5, 6]. Several studies have been reported on selected liver function abnormalities and mechanisms of these abnormalities include generalized inflammation, vasculitis of small and medium sized vessels, congestive heart failure secondary to myocarditis, toxin-mediated effect of nonsteroidal anti-inflammatory antipyretics, and a combination of these events [1–4, 7].

We report a case of KD with cholestasis as principal symptom in patients admitted for fever.

## Case presentation

A previously healthy, blood donor, 43-year-old caucasian man was admitted to our Internal Medicine ward because of fever (38.5 °C), posterior chest pain, abdominal pain, and earache.

Three days earlier, he was seen in the emergency room for fever and pain in his right ear. External otitis was diagnosed and he was discharged home on oral amoxicillin-clavulanate (1000 mg tid) and paracetamol treatment. On that occasion laboratory tests showed increasing C-reactive protein (CRP 6.93 mg/dl) and alanine aminotransferase (ALT 623 U/L).

The patient was sent again to the hospital by the general practitioner for admission due to the persistence of fever (max value 41 °C), abdominal pain in the right hypochondrium and the appearance of jaundice with hypocolic faeces and hyperchromic urine. He was admitted to the internal medicine ward. At the time of admission, physical examination showed jaundice and presence of some bilateral laterocervical lymph nodes, mobile compared to the underlying planes. No other alterations were detected.

Blood tests revealed increased white blood cells count, CRP was 5.8 mg/dl, total bilirubin 5.88 mg/dl, direct bilirubin 3.42 mg/dl, there was moderate increase in lipase (255 U/L), while ALT was 161 U/L. Moreover, serology for HBV indicated a previous vaccination, while serology for HCV, Herpes Simplex Virus, Citomegalovirus, Herpes Zoster Virus, Parvovirus B19, Bartonella, Leptospira, Coxsackie, Monotest and Toxoplasma and other hepatitic virus (HDV and HEV) were negative for current infection.

Chest X-ray and abdominal ultrasound were normal. Subsequently a chest and abdominal CT and magnetic resonance were performed which did not show changes in the liver, biliary tract or pancreas, but only the presence of some mediastinal lymph nodes (diameter 11–13 mm) in axillary bilaterally, abdominal area of the omentum, retroperitoneal cavity, and along aorta and iliac vessels bilaterally. Electrocardiograms were always normal as well as echocardiography.

Amoxicillin-Clavulanate and paracetamol were stopped, thinking about iatrogenic liver toxicity and replaced with ceftriaxone and metronidazole. Subsequently, due to persistent fever and elevated ESR and CRP, Piperacillin-Tazobactam and Tigecycline were given. On 4th day of hospitalization total serum bilirubin increased up to 12.06 mg/dl and direct bilirubin was 7.07 mg/dl. Bilateral non-exudative conjuntival injection (red eyes) was still present.

On 6th day of admission, the patient's condition worsened with hyperpyrexia, very deep asthenia, arterial hypotension, tachycardia, anaemia and multiorgan failure. Patient was admitted to the Intensive Care Unit, and clinical condition was supported with oxygen supplementation, fluids, nutritional support, antibiotic therapy (Meropenem, Fluconazole) and Methylprednisolon.

The autoimmune profile work-up and lupus anticoagulant, Weil-Felix, Vidal-Wright reactions and examination for plasmodium falciparum malaria were negative. Immunoglobulin values and lymphocytic typing were normal. Plasma IgG4 dosage resulted within the limits. The stool culture, including Clostridium Difficile and Cryptosporidium, parasites and eggs on faeces were negative.

On 10th day of admission, hepatic biopsy found a fragmented liver tissue with accentuated lobular architecture, representative of portals enlarged for sclerosis and lymphocytic inflammatory infiltration, macrophage and mainly granulocyte, with involvement of the lobule and aspects of bile ductal proliferation. Presence in the lobule of numerous inflammatory infiltrates with a predominant neutrophilic granulocyte component with foci of hepatocytolysis, acidophilic bodies and occasional biliary thrombi were detected. Moreover, modest hepatocyte and hemosiderin deposits were observed.

**Fig. 1:**
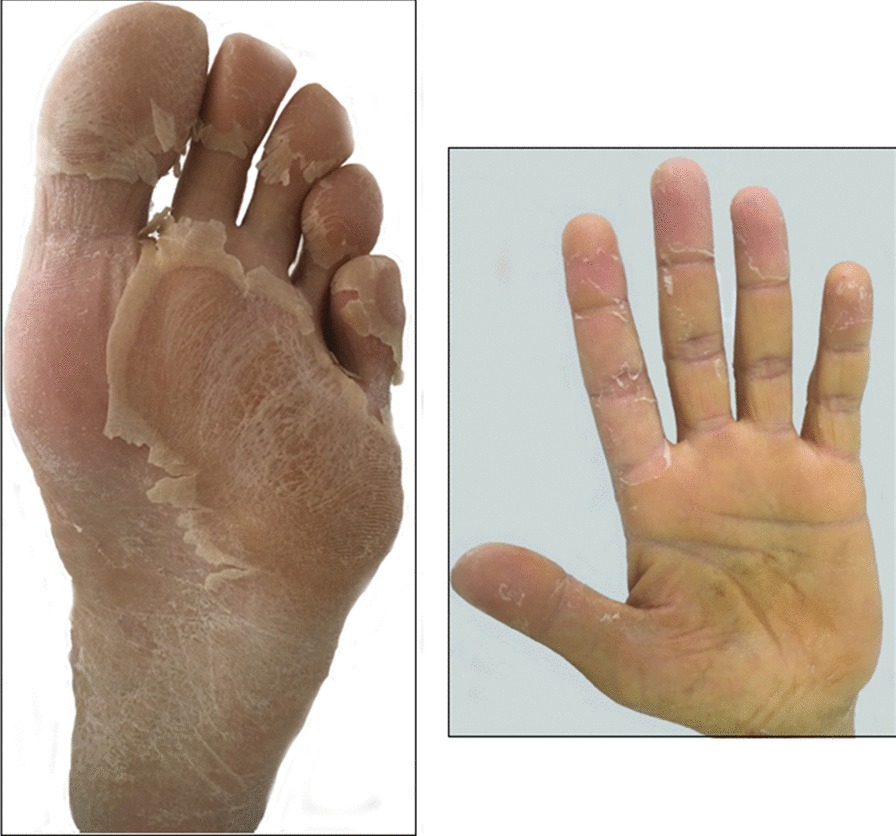
Lamellar desquamation of the palms and foot on 22th day of hospitalization

**Fig. 2:**
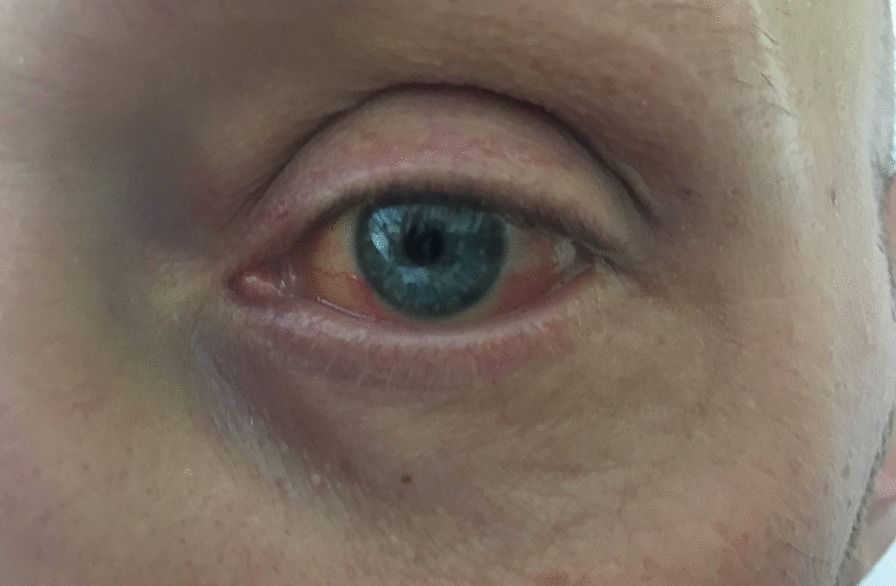
Slight desquamation of the face bilateral and non-essudative conjuntival injection on 18th day of hospitalization

On 13th day of hospitalization, cardiovascular conditions improved, but temperature was still high on 15th day of hospitalization, while cholestasis parameters began to reduce, and patient was sent back to internal medicine ward. The patient was profoundly asthenic (so much so that he could not even sit in the bed), dysphonic, febrile (38.5–39 °C), he had jaundice, and presented small bilateral laterocervical lymph nodes not painful. Furthermore, bilateral non-exudative conjuntival injection and slight desquamation of the face (Figs. 1, 2), lip cracks, dryness and lamellar desquamation of the palms of the hands and feet were present; in the following day the peeling of the hands and feet progressed dramatically (Fig. 2).

**Fig. 3:**
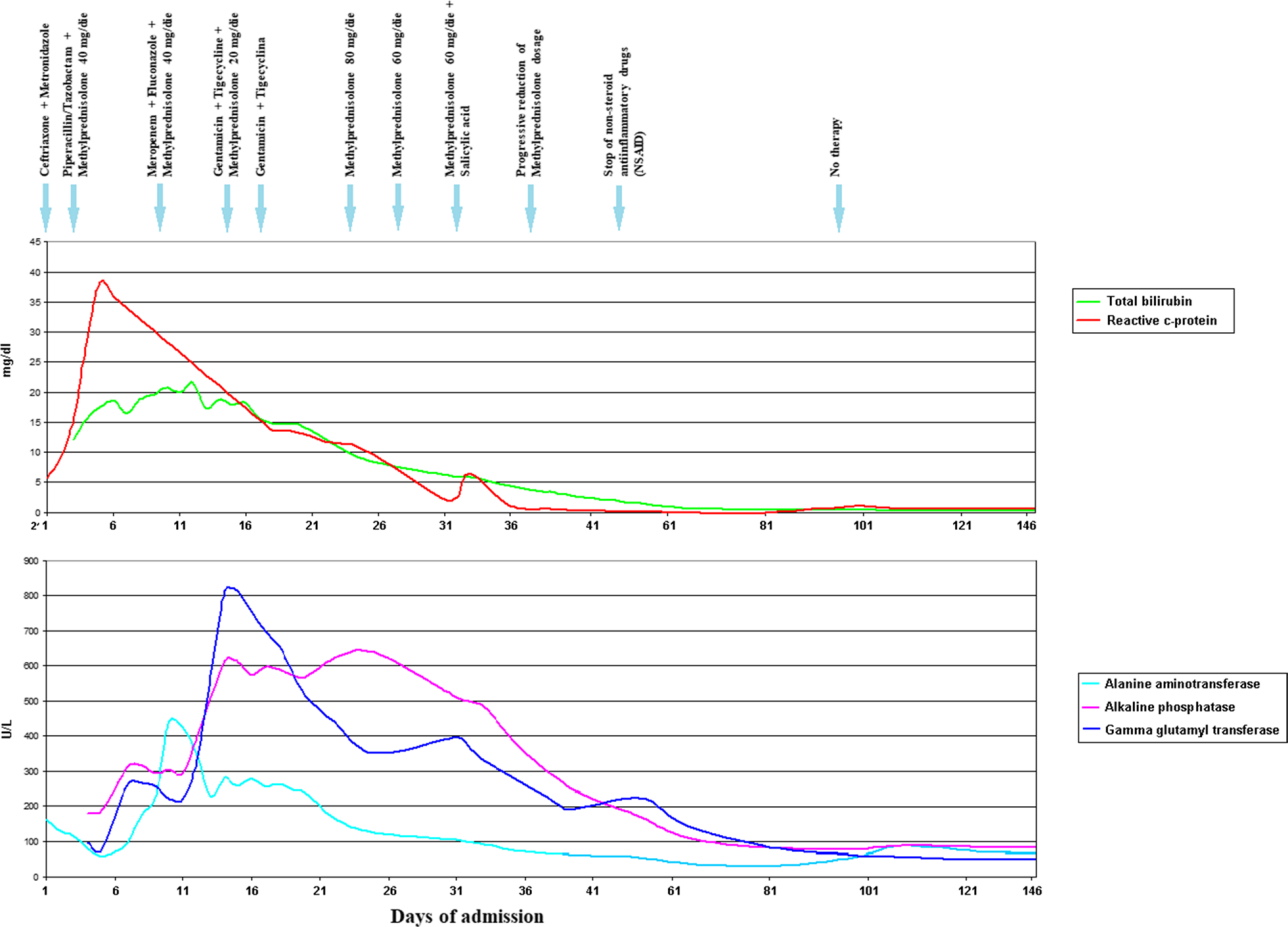
Patient’s biochemical tests (total bilirubin, c-reaction protein, alanine aminotransferase, aspartate aminotransferase, gamma-glutamyl transpeptidase, and treatment during hospitalization

On 24th day of admission, due to the persistence of jaundice, asthenia, dysphonia, red eyes, corticosteroid therapy (Methylprednisolon 80 mg/die) was set, instead of antibiotic therapy due to the persistence of negative culture tests. On the following days the fever rapidly disappeared, and the patient reported net improvement of asthenia and anorexia, and a clear reduction in conjunctival reddening. Laboratory tests showed reduction of acute phase reactants, and progressively also of cholestasis (Fig. 3). He also underwent arterial doppler of the lower limbs that was unremarkable.

Starting from the 28th day of admission, corticosteroid therapy was progressively reduced and intravenous lysine acetylsalicylate 2000 mg/die was given. Jaundice (total bilirubin 6.23 and direct bilirubin 2.79 mg/dl) and parameters of hepatic cytolysis (alanine aminotransferase 104 U/L, gamma-glutamyl transpeptidase 396 U/L) improved.

At 37th day of hospitalization, corticosteroid therapy and lysine acetylsalicylate were switched orally.

The patient kept on improving. He was gradually able to get out of bed and within a few days he was able to walk initially helped and then independently. Blood exams also improved with normalization of white blood cells, CRP and total bilirubin decreased to 3.31 mg/dl.

After 40 days of hospitalization, the patient was discharged tapering corticosteroid therapy of 4 mg every 3 days.

On the 47th day after the hospitalization he performed a Coronary Computed Tomography Angiography which was normal. Three weeks after discharge he managed to resume his work. Eighteen months later the patient was completely asymptomatic.

## Discussion and conclusion

The KD's aetiology is still unknown, and has been hypothesized that it develops in the genetically predisposed patients due to a possibly infectious trigger.

The diagnosis of KD relies on clinical signs and symptoms since no specific diagnostic tests are available. In the classic form the patients are children, and they have fever for more than 5 days and at least 4 out of 5 diagnosis criteria [1–4]:

- oral lesions such as erythema of oral and pharyngeal mucosa, cracking or fissured lips, strawberry tongue;
- bilateral conjunctivitis without exudate;
- polymorphous rash;
- oedema or erythema of the extremity with a progressing to desquamation of feet and hands;
- cervical lymphadenopathy usually unilateral (≥ 1.5 cm)

The patient we studied presented 3 of these criteria however, some patients show only a few clinical features of the typical clinical picture of KD, defining as incomplete form of KD often underdiagnosed [5] especially in adults [4].

The low diagnostic accuracy for adults onset of KD could be attributed to the several confounding factors [1–5, 8–10] such as drug hypersensitivity reactions, toxic shock syndrome, erythema multiforme and Stevens-Johnson syndrome, Scarlet fever, Measles, Rubella, infections to Adenovirus, Parvovirus, Herpesvirus, Epstein-Barr virus, Toxoplasmosis, Leptospirosis, Rocky Mountain spotted fever, Systemic juvenile-onset idiopathic arthritis, rheumatic fever, Reiter syndrome, and mercury poisoning.

Cervical adenopathy, hepatitis and arthralgia are found more common in adults (93%, 65% and 61%, respectively) than in children (15%, 10% and 24–38%, respectively). Children can suffer more frequently of meningitis, thrombocytosis and coronary artery aneurysms than adults (34% vs. 10%, 100% vs. 55%, 18–25% vs. 5%, respectively) [1–6, 10].

Less common symptoms of KD include arthritis, jaundice, hepatitis, myocarditis or heart failure, pericarditis, diarrhoea, anterior uveitis, headache and aseptic meningitis [1–6].

Common laboratory changes include raised acute phase reactants, hepatic cytolysis, delayed thrombocytosis, hyponatremia and acute kidney injury, eosinophilia and aseptic leukocyturia [6, 10–12].

In the present case, we could not detect drug hypersensitivity reactions, toxic shock syndrome, or infectious diseases, and diagnosis of KD was suggested by three well-accepted criteria for diagnosis. However, unusual clinical features were noted:

1. prevalence of KD is much higher in Japan than in Europe, especially in children;
2. the disease occurred in a young adult;
3. abdominal complaints were particularly severe and represented the cause of hospitalization;
4. jaundice was the principal symptom;
5. any cardiovascular involvement was not detected.

Demography, atypical presentation, rapid development of multiorgan failure were the main reasons for delaying diagnosis. Moreover, jaundice could had masked any other cutaneous erythematous manifestation such as polymorphous rash.

In adults with KD gastrointestinal involvement has been reported in 56% of patients, abdominal pain (20–26% of patients), emesis (14%), diarrhea (12–20%), hepatomegaly (14%), jaundice (23%), and splenomegaly (12%) have been described [6, 10, 18, 19].

Hepatobiliary involvement is uncommon in Kawasaki disease, but it is usually described as obstructive jaundice [20]. Abnormalities of liver panel have been documented in 30–45% of cases [10, 18, 19, 21].

This presentation may act as a confounding factor leading to a delay in diagnosis and subsequent treatment [21].

Liver involvement could range from increasing in liver enzymes to a severe cholestatic hepatitis and/or gallbladder hydrops [21, 22].

Obstructive jaundice with hepatic dysfunction due to gallbladder hydrops has been reported in around 13% of case, however being asymptomatic the condition could be more frequent than previously reported [18–21]. In the majority of cases, hydrops resolves spontaneously without requiring surgical intervention [18–21]. On the contrary, when laparotomy exploration is performed, it reveals inflamed and edematous gallbladder without stones, and hypertrophic mesenteric lymph nodes. In 1979, it was reported that vascular lesions in KD were histologically similar to systemic vasculitis [23].

As well as in our case, an acute episode of cholestasis, with normal gallbladder and bile ducts preceding general manifestations of KD, has been reported in a 9-year old girl, [24]. Moreover, a 16 years old boy with KD presented with fever, jaundice, and abdominal pain [25].

The mechanism of painful jaundice that occurred in our patient could be related to a distention of the gallbladder, but it spontaneously resolved at the time of ultrasound examination. A vasculitic process in the gallbladder could be supposed to be the cause of an inflammation of the serosa and of obstruction [20]. Abdominal CT in our patient showed enlarged lymph nodes.

In our case, hepatic biopsy found non-specific inflammatory alterations in a particular fragmented liver tissue with accentuated lobular architecture, and lymphocytic inflammatory infiltration, macrophage and mainly granulocyte, several inflammatory infiltrates with a predominant neutrophilic granulocyte component were observed with foci of hepatocytolysis, acidophilic bodies and occasional biliary thrombi. These findings are similar to those described in KD patients with hepatic presentation or in autopsies [18, 20, 26, 27].

Ideal treatment of KD for a child should be administration of both intravenous immunoglobulins and acetylsalicylic acid.

Intravenous immunoglobulins reduce cardiac complications such as myocardial infarction and sudden death [3, 13]; however, the exact mechanisms is not clear, probably immunoglobulins influence T cell differentiation.

Acetylsalicylic acid appears to act on the endothelium, which is known to be dysfunctional in KD. Both Joint Working Group (JCS) and American Heart Association (AHA) [4, 14] recommend antiplatelet agents, primarily acetylsalicylic acid, during the acute phase of disease until aneurysmal pseudo-normalization. The 2017 AHA guidelines note that dual antiplatelet therapy (DAPT) may be considered in some cases, though there are no studies evaluating the efficacy of DAPT in this context.

Nevertheless, in KD acetylsalicylic acid was not reported to reduce development of coronary abnormalities [15].

Therapy in the acute stage of KD includes acetylsalicylic acid in combination with high-dose intravenous immunoglobulins. This treatment could reduce the prevalence of coronary abnormalities, the most serious complication of this disease, but it also favors a rapid reduction of fever and normalization of acute phase reactants.

Corticosteroid treatment appears to be beneficial in patients with severe KD, showing resistance to intravenous immunoglobulins. Combination of corticosteroids and intravenous immunoglobulins is indicated in patients at high risk of unresponsiveness [3, 16, 17].

In the present case intravenous immunoglobulins were not used, because of uncommon presentation, diagnostic delay, lack of experience in similar cases and, above all, because of absence of myocardial and vascular involvement.

Corticosteroid and acetyl salicylic acid were given due to the persistence of cholestasis, high acute phase reactants plasma levels and histological findings.

Our case suggests that atypical KD could be added to the etiological list of painful febrile jaundice in young patients. Physicians should pay attention to the diagnosis of atypical KD in the middle-aged persons and take action as soon as possible to avoid deadly complications.

While adult-onset Kawasaki disease is rare, clinicians should be familiar with the diagnostic criteria to consider them in the differential diagnosis of patients presenting undifferentiated febrile illness. Early treatment should be prescribed to prevent sequelae.

## Data Availability

The datasets supporting the conclusions of this article are included within the article.

## Abbreviations

KD: Kawasaki disease
AKD: Adult-onset KD
